# Research on Papua, a digital tool with artificial intelligence in favor of learning and linguistic attitudes towards the learning of the English language in students of Spanish language as L1

**DOI:** 10.3389/fpsyg.2022.1019278

**Published:** 2022-10-31

**Authors:** Beatriz Peña-Acuña, Rafael Crismán-Pérez

**Affiliations:** ^1^Facultad de Educación, Psicología y Ciencias del Deporte, Universidad de Huelva, Huelva, Spain; ^2^Departamento de Filología, Facultad de Filosofía y Letras, Universidad de Cádiz, Cádiz, Spain

**Keywords:** language learning, English language, study and teaching, foreign speakers, digital analog conversion, Spanish language, attitudes, inclusive education

## Abstract

This study examines learners’ perceptions of the linguistic and learning potential of an AI-based English language learning app called Papua. The study took a quasi-experimental approach in which 128 students of the degree in Primary Education at the University of Huelva, Spain, gained experience of using the app over a six-week period, and then answered a questionnaire. This was of a quantitative design, and included the following components: attitudes towards learning English; attitudes towards learning oral and written skills, and towards opportunities for interacting in English; and attitudes towards the motivation provided by the app. These attitudes were contrasted according to the variables memory and self-evaluation. The results of the study showed that oral skills were perceived as the most improved skill. Participants also foregrounded the enhancement of memorization of vocabulary, and positively evaluated the self-evaluation feature included in the app.

## Introduction

Among the range of foreign language learning options, Computer Assisted Language Learning (CALL) has seen good results (Jen-Jiun and Huifen, 2019; Khafaga and Alghawli, 2021). A recent development in this kind of software is the use of apps, which have become increasingly popular as a medium of instruction in general (García-Aretio, 2016), as well as specifically for learning English, with the incorporation of artificial intelligence (Jin et al., 2019). Such programs incorporate a range of approaches, such as task scaffolding and conversational interaction (Chie Fang et al., 2021), and the development of speaking (Moreno-Ibañez et al., 2016), reading and writing (Liou, 2016) skills. One of novel features of these apps currently being researched is the inclusion of dynamic assessment (henceforth DA), by which the learner takes an active role (Rezaee et al., 2019).

Mobile Assisted Language Learning (MALL) is breaking new ground in the area of research into the process of language teaching and learning. In one study, listening comprehension skills were found to be a more effectively developed when learners watched the movements of their own 3D avatar in Second Life as opposed to performing the same movements themselves or following the task without making any movements (Lan et al., 2018). This study is just one example of the multiple possibilities which have been opened up within the field of language education by these programs in recent years. Research into MALL has taken various methodological approaches and considered many different variables, among which can be found quantitative (Lin and Lin, 2019), qualitative (Luef et al., 2020) and mixed methods (Al-Ahdal and Abdullah Alharbi, 2021) studies. The majority of the studies report positive findings regarding the effectiveness of such apps (Rezaee et al., 2019).

The use of technology in education was already widely established, including Artificial Intelligence, but its full implementation has been accelerated by the circumstances of the pandemic and by the tendency to personalize Apps according to studies such as that of Parra-Sánchez (2022). It is not so much an advance in Artificial Intelligence but in the massive use of technologies in sectors of society where they had not penetrated so much before. For example, virtual glasses already existed as Nintendo’s Virtual Boy released in 1995, but it is not until now that their use has become widespread. In education, in adolescents and post-adolescents, there is an overuse at times of mobile phones and social networks, so much so that, for example, it has become a good way of promoting the reading habit, as stated by Hernández Ortega et al. (2021). In this sense, Artificial Intelligence comes to take its place within the massive use of technologies, in societies that can afford it.

The app we investigated, Papua, is an artificial intelligence-based application which incorporates several functions, including voice recognition and pronunciation coaching. It is aimed at developing spoken fluency, reading and writing skills, grammar and socio-cultural skills. Its main features are the following:

- Instructional immediacy and ubiquitous learning. This means learners can access the app wherever they happen to be and so maximize the use of their time.
- Compatibility with most devices – smartphones and tablets – so that learners can easily connect and interact with the interface.
- Teacher supervision. The app allows teachers to supervise and evaluate their learners’ quantifiable progress *via* the platform on the PAPUA website.
- Tools for receiving support and feedback on progress. The app scaffolds user learning when necessary, breaking it up into more manageable units (García Botero et al., 2021). Users access the website *via* a password, after which they can receive evaluative input from the teacher along with feedback on their performance through DA.

All these novelties are underpinned by the software which enables the device to give the learner immediate feedback. From the user perspective, the app is structured according to a series of scenarios following a storyline. The user takes the role of protagonist in these scenarios, and is offered a set of alternatives such that the story develops dynamically in response to the user’s choices. The user can select both the degree of difficulty and the itinerary. The app dynamically updates these choices in real time to move the learner through the succession of different learning scenarios. At the end of each itinerary, the app produces a set of final results according the user’s responses along the way. These results include pronunciation (the phonetic-phonological dimension of language use), grammar (the morpho-syntactic dimension), vocabulary (the lexical and semantic dimension) and cultural issues and references (the pragmatic dimension).

## Research on language learning app with IA

This study pertains to the relatively recent field of study into the effectiveness of apps. It evaluates an artificial intelligence-based app offering a series of specific features. It aims to assess the effectiveness of two aspects of the app, with the following specific objectives. First, find out users’ recognition of the cognitive impact on memory. Second, find out users’ perception regarding the effectiveness of the self-evaluation provided by the app. So, this paper explores users’ appreciation of two of these aspects which previously have not received much attention, specifically learners’ memorization of content (Al-Ahdal and Abdullah Alharbi, 2021), and management of evaluation a form of self-regulation, as understood by García Botero et al. (2021).

Both these dimensions were explored in this study of university undergraduates learning English as a foreign language. Memory turned out to be a decisive variable with respect to gender, age, and the students’ level of study (Wang et al., 2021). In addition, the learning strategies based on memory were found to be more stable among the students participating in the study.

At the same time, we took self-evaluation into account as the second research dimension, among other reasons, because of the current boom in this kind of methodological approach to evaluation, and because of the implications it could have for other variables of an affective nature (García Naranjo, 2018; Andrade, 2019; García Sanz, 2020).

In monitoring these two dimensions, we aimed to cover both direct and indirect strategies involved in language learning (Oxford, 1990). Hence, with respect to direct strategies (memory strategies, cognitive strategies and compensation strategies), memory was considered the chief factor in the study, whilst in the case of the indirect strategies (social strategies, affective strategies and metacognitive strategies), we took self-evaluation into account for its affective and metacognitive connotations as a general methodological strategy in language learning.

Beyond the novelty of monitoring these two combined dimensions in university level EFL students, the main contribution of this study lies in the analysis of these strategies in relation to the use of the Papua virtual reality tool for learning English. This meant observing the strategies operating in the context of a virtual learning methodology. In the last few years, this kind of methodology has become extremely popular, to the extent that many researchers have expressed the need to further develop learning tools of this kind (Chu-Chu et al., 2013; Santamaría and Alcalde, 2021). For this reason, we took SE into consideration as a potential means of improvement in this process in comparison with other MALL applications.

### Memory

As Huang et al. (2022) note, memory has traditionally been considered one of the four components of linguistic aptitude (Carroll, 1981) for learning first and second languages, alongside phonemic coding ability, grammatical sensitivity, and the ability to learn inductively. Specifically, associative memory – the ability to make connections between stimuli and stored responses – was long considered to be a major factor in language learning, but working memory has come to supplant it as the key aspect of memory in this area (Skehan, 2015). Working memory is linked to complex cognitive tasks such as understanding language, learning and reasoning. Through these tasks the system not only understands and manages stored information, but also stores and processes the information temporarily at the same time.

Working memory is thus connected to the central executive system, attention control, and three subordinate systems, namely the phonological, visual spatial information, and episodic memory. All this is what allows working memory to be able to simultaneously store information and process it in context. This processing implies that the content stored in working memory has been through a selection process, given the huge amount of sensory information the individual is constantly receiving. In this way, working memory operates with conscious information, while at the same time there is potentially manageable virtual memory. This duality between stored information and potentially processable information, along with its link to other higher cognitive tasks, turns out to be vitally important in the context of EFL/ESOL learning/acquisition (Shen and Park, 2020). As the two authors note (2020, p. 86):

According to this model (Baddeley, 2000), working memory is comprised of four components which process distinct types of input information. Auditory information is stored and processed by the phonological loop, while the visuospatial sketchpad is responsible for visual images and spatial relations. The central executive plays a role similar to a traffic cop who regulates the other components of working memory and allocates cognitive resources. The episodic buffer is assumed to be the storage component of the central executive, and a link from working memory to long-term memory. In the information processing model, long-term memory is the final stage.

#### Working memory

Working memory is of paramount importance, due, among other factors, to the constant feedback between the phonemic coding/decoding and the visual spatial management of alternative learning scenarios. Especially so in the case of the app in question, Papua, as said learning scenarios are virtually unpredictable, as they are the result of the prior choice of difficulty level on the part of the user, and their responses during the interactive leaching-learning process. In consequence, the attentional component plays a major role in the learning process, given the constantly changing nature of the scenarios and situations of said learning contexts.

The overlap of learning scenarios leads to the need to handle a greater quantity and wider variety of information, which can potentially be stored in long-term memory after first being selected by working memory. Consequently, the memorization of information can be increased with this kind of virtual tool for the learning/acquisition of a foreign or second language. In this regard, this study considered that the Papua virtual tool would improve these memory-related strategies. The students needed both declarative and working memory in order to complete the activities included in the app. The study by Al-Ahdal and Abdullah Alharbi (2021) provides an appropriate starting point. These researchers showed that the collaborative use of mobile devices by the experimental group improved their retention of vocabulary post-intervention, while overall group performance drastically improved, with more learners scoring closer to the mean value. The control group, by contrast, showed no significant difference in performance.

The first research question forming the basis of this study was the following: Does Papua improve users’ vocabulary retention as a result of its interactivity and constant feedback on their responses? This feedback relates to the possibilities for storage of content by students and the operative possibilities for simultaneous processing of this information by the subjects of the sample. Both tasks are linked to the users’ working memory.

#### Storytelling and memory management

At this point, we should also highlight the concept of storytelling as a thematic thread running through the interaction between the user as protagonist and the Papua app as another influential factor in memory management.

As McGregor and Holmes (1999) note, the concept of storytelling boosts long-term memory in comparison with other narrative techniques. Secondary cognitive effects are produced through storytelling techniques, and these contribute to idealization and satisfaction in relationships, which in turn converge on the quality of the foreign/s language learning/acquisition process by virtue of affect (Arnold, 2000).

In similar vein, the concept of storytelling, as adopted by Papua, draws on the so-called *enactment effect*, also known as the *self-performed task effect*. The effect basically refers to the fact that a linguistic sequence represented by an action is memorized better than a linguistic sequence without such representation, especially if the learner physically enacts the sequence virtually. This representation of action has an effect on action memory (Zhang and Zuber, 2020).

In our approach, it was expected that these novelties in Papua would contribute to an improvement in the quality of the learning process in terms of vocabulary retention as a result of the development of working memory on the part of the user, who has to simultaneously process the stored contextual information. Relevant studies in this regard include the traditional use of narratives in the primary classroom (Kirsch, 2016), and its use as a teaching strategy in language learning (Rezende, 2016). These studies found positive results for storytelling techniques applied to learning languages.

The present study builds on these previous studies with the aim of verifying the degree of success of the storytelling concept for learning English with two new variables: the sample was composed of adult university education students, and the storytelling element was carried out in a virtual interactive environment. In place of a physical storyteller, Papua uses an action framework in which users visualize themselves in a learning scenario requiring a series of actions related to learning English to be carried out.

This approach represented a step forward in internalizing content. Rather than visualizing an external agent for carrying out the action, or assimilating content in an abstract manner, users themselves carry out the activities, thus reinforcing retention.

Further, for verifying the success in learning/acquiring vocabulary, the Papua app structured strategies involving the repetition of words and expressions by the user according to a variety of sentence and sub-sentence-based schemes inserted in certain communicative situations, at the same time the same program pronounced a series of words and expressions chiefly by designation. The program, through said strategies for the learning/acquisition of EFL/TESOL, featured a function for correcting the user’s pronunciation while it offered a rating as a result of a process of evaluating the quality of the pronunciation (which was also extendable to other functions and modules, such as lexis, through the evaluation of vocabulary and grammar retention).

These strategies resulted in simultaneously enhancing vocabulary retention and improving pronunciation and comprehension of English as foreign or second language (thus linking to the oral skills of speaking and listening).

This derives from the fact that working memory, as mentioned above, took on a major role in the interactive learning/acquisition process between the students and the software, which gave rise to the processes of phonetic coding and decoding being reinforced in comparison with other programs and tools for learning a foreign or second language. Previous studies of relevance include those (Han, 2015; Han et al., 2017; Wang, 2019). These studies found a correlation between the working memory and oral skills of Chinese learners of English across a range of ages, with a stronger correlation of working memory with oral production than with writing skills. Nevertheless, working memory was found not to have such significance in relation the written skills according to other researchers into English language learning with Chinese students (Yi and Luo, 2012; Yi and Ni, 2015). In these studies, aspects such as motivation and self-regulation in the language learning process were taken into account. This led us to take into consideration, in addition to the memory component, self-evaluation as a process in monitoring one’s actions and their outcomes.

Additionally, one of the unique features of the Papua tool is the possibility of real-time narrative-based interaction in the form of a story. Through this feature, users can choose from various response options which entail corresponding changes in the way the action unfolds. We thus consider that memory represents one of the fundamental element for students, as the choices they make will converge in parallel stories. This means working on the two main dimensions of memory: declarative and procedural. Likewise, the possibility of intervening in real time in the story enhances the general constructivist principle of the protagonist role of the learner during the learning process.

### Evaluative feedback from the app

Papua provides feedback on user performance across a range of scales corresponding to various aspects. To this extent, the process constitutes a form of self-evaluation (SE), a common practice in educational settings in different domains. The teacher can also access the student’s progress on the website platform by tracking the textual indicators providing the specific feedback. This metacognitive tool allows students to gauge their progress (Rodríguez Gómez et al., 2009; Fernández-Novell and Zaragoza Domeneq, 2014), and to gain autonomy (Fernández, 2011). SE has especially been taken up in language education (García Botero et al., 2021), and has been received positively by education graduates as a means of increasing participation (García Botero et al., 2019), although it has been criticized elsewhere.

Calatayud (2002, p. 358) defines SE as *the foremost strategy for developing responsibility and for learning to value, critically assess and reflect on the process of individual teaching and learning*. *It concerns the knowledge of what has, and has not, been achieved towards gaining this command, and what further steps can be taken to correct errors and improve the outcomes*. García Botero et al. (2021), p. 41 notes that learning strategies are acquired through SE: *SE is not only the evaluation of the learner’s own performance or the ability to make judgements about their own performance, but rather a means of developing strategies for learning and for improving learning outcomes*.

With respect to Papua, the SE adopted falls within the framework of *Mobile-based Dynamic Assessment* (MDA) in which the student has a positive attitude to language learning. It provides users with detailed information on quantitative scales featuring two measures focusing on positive feedback and three on negative, in the following five areas:

- Pronunciation score. This function awards the learner a score for the accuracy of their pronunciation in English. 100% is a perfect score.
- Response score. This function also gives the learner a score out of 100 for the speed and fluency of their response.
- Wrong answers (content). This item gives the learner negative reinforcement according to behaviorism model.
- Wrong answers (grammar). This item gives the learner negative reinforcement according to behaviorism paradigm.
- Wrong answers (socio-cultural competence). This item gives the learner negative reinforcement according to behaviorism theory.

The effect of MDA on students’ EFL fluency was investigated by Rezaee et al. (2019). Analysis of the results showed that the experimental groups – that is, those receiving MDA – performed significantly better than the control group in the post-test. The findings of the study suggested that MDA had a positive effect on the EFL learners’ oral fluency. Hence, after considering the findings of these previous studies, we hypothesized that the constant feedback provided for the students by the app would improve their strategies for learning English as a result of mainly their indirect strategies being enhanced and the leading role of the learner (Oxford, 1990).

In similar research, García Botero et al. (2021) carried out a study into self-regulation and scaffolding for supporting out-of-class MALL. One of the two experimental groups received training in self-regulation and scaffolding support in preparation for their MALL experience. The results of the study suggested that this training was beneficial for student uptake of MALL, along with several other benefits. Those students who were trained in self-regulation and received temporary scaffolding demonstrated higher levels of participation and greater motivation than those who received no preparatory training. The study found a significant link between self-regulation and intrinsic motivation.

On the other hand, we think that the opportunity for self-evaluation, in addition to being motivating, might represent a significant factor in affective involvement in the learning process, as it provides their avatar in the app with real choices that the students must make. The projection of the learner from the avatar allows the student to monitor their own learning process in real time. This provides greater autonomy to it with respect to its evolution and, therefore, may have implications in factors related to the affective and cognitive dimension of the learner’s attitudes by virtue of their motivation according to a possible link between autonomy and self-regulation of the process of learning/acquisition of foreign languages/s languages.

#### Intrinsic motivation

Intrinsic motivation, a psychological construct, refers to the personal satisfaction inherent in an activity which leads to the engagement and enjoyment of the person involved. Intrinsic motivation thus constitutes an end in itself (Deci and Ryan, 2000). This has currently been corroborated by studies with positive results into self-regulated learning (Wang, 2019; Chih-Ming et al., 2019).

Likewise, there have been various positive studies into how language learning apps can draw on intrinsic motivation using MBA.

Cahyono and Ludwig (2017) indicated that intrinsic motivation and identified regulation became essential factors in the students’ motivation to participate in the activity. Jeno et al. (2020) found a credible interaction effect between the mobile app and intrinsic goal-framing for intentions and identified regulation. For the variables *effort* and *achievement*, the main effect of mobile learning was consistent with substantial effect sizes.

Chin-Hsuan et al. (2021) indicate that conscientiousness and m-learning readiness are critical antecedents of intrinsic motivation and extrinsic motivation. In addition, both types of motivation had a significant positive effect on students’ intention to use this kind of app in terms of encouraging progress through the language learning process.

#### Promoting progress and personalized learning

According to (Pérez Ariza and Hernández Sánchez, 2014), the question of progress in learning has been the subject of detailed study within the fields of both psychology and education, with valuable contributions by internationally renowned theorists such as Thordinke (1931), Piaget (1972), Bruner (2001), Vigotski (1987) and David et al. (1991). These authors affirm that any learning process presupposes some kind of cognitive transformation in the individual, such that any process concerns its own development.

In the field of educational technology, automatized digital tools have been designed to promote learning and, in some cases, provide feedback. For example, Charles (2009) filed a patent for a automatized device for language learning which draws on specific content from a database, combining this information with images for viewing. It also promoted active listening on the part of the user. Other devices, such as the system created by Kao et al. (2009), evaluate learning progress in the following manner. The learning module plans lessons according to the progress measured by the test module. An evaluation module sets the evaluation for the lessons covered and provides feedback with the aim of evaluating user progress. The progress test questions are stored in a database along with the responses, so that these can be considered in future tests.

García Botero et al. (2019) carried out a study comparing student SEs with the assessment descriptors provided in an oral communication skills course reaching the conclusion that students require guidance in carrying out SE activities. The study also noted the need to pay special attention to the wording of the SE grid used by the students to assess their learning, given that few students used this wording in their self-reports, and instead either mixed the given wording with their own expressions, or relied entirely on their own wording. It was also notable that the perceived importance of particular areas of learning did not match the self-evaluated progress across the same range. All these tools take into account individualization and personalization in the process of learning a foreign language.

Personalized learning is an area which has been considered by educators and teaching professionals at both the theoretical and empirical levels (Fernando Calderero et al., 2014). Over the last few decades, the approach has become an established paradigm applied across a range of educational contexts. According to the authors (Fernando Calderero et al., 2014, pp. 140–141), it is characterized by three main features: uniqueness, autonomy and openness. In the definition provided by García-Hoz (1988, p. 25), ‘personalized education is personalized to the extent that it is applied to an individual with their own unique set of characteristics, who feels engaged and committed to realizing their personal possibilities, and who, at the same time, is ennobled by the mere fact of being and doing as a person.’

Educational technology is also directing itself towards personalized learning, with various patents being filed to this effect. An example is Chao et al. (2009), which describes an intelligent, interactive learning system. The system automatically generates learning materials by capturing and restructuring information from the internet. By tracking users’ learning behavior it is able to provide personalized materials more efficiently. According to Zhou et al. (2016), among current areas of research into personalized learning can be found data-mining, massive open online courses (MOOC), the learning environment, learning analysis, ubiquitous learning, learning styles, E-learning, and teaching modes. In like fashion, research into personalized learning models has tended chiefly towards exploring theoretical frameworks, diversification, and the implementation of personalized modes and technology.

## Aims

The general aim of this research paper is to evaluate ‘Papua’, an app designed to facilitate learning English, whether as a foreign (EFL) or second (ESL) language. The app downloads onto any portable device (smartphone or tablet), and is the product of a start-up called Aprendizaje Inmersivo S.L. (Immersive Learning Ltd.), based in Mósteles (Madrid). It has been developed by a multi-disciplinary, multinational team, and uses artificial intelligence to develop different communicative competences.

The specific aim is to evaluate the efficacy of the app in two dimensions: memory reinforcement and self-evaluation from the process of self-regulation of learning/acquisition of English as a foreign language.

### Research questions

From all the above, this study considers four research questions:

1. What impact does the virtual tool Papua have on memory-based strategies with respect to university level learners of English as a foreign language?
2. What impact does the self-evaluation methodology of the virtual tool Papua have on the English language learning of university level students?
3. Does the intervention program through the Papua app improve the oral skills of the students comprising the sample more than their written skills?
4. Did the incorporation of the components *intrinsic motivation*, *monitoring of progress*, and *personalized learning* into the SE function of the Papua app, have a significant impact on the language learning process?

## Materials and methods

### Participants

We recruited a sample of students in the second year of a degree in Education in the academic year 2020–2021, who participated in a quasi-experimental study meanwhile they followed an Education Degree in the university level. In the case of these Spaniards, the students had a particular interest in participating in the study as, in order to graduate, they were required to accredit a level at B1 (as specified by the Common European Framework of Reference) in any of the official languages of the EU by means of certification through one of the many recognized examination bodies, although for the purposes of this study the language selected was English. This stipulation is a recommendation by the Council of Europe, take-up of which has not been uniform either across the EU or indeed within Spain itself, where devolved responsibilities have seen significant variation across autonomous communities in terms of implementation and required level, Andalusia (where the study took place) currently opting to implement the recommendation at B1. Given its status as a *lingua franca*, English is the predominant second language choice, as achieving the stipulated level not only meets their degree requirements, but also provides students with a necessary competence in the labor market.

The sample consisted of 128 students from two groups in their second year of the degree in Primary Education at the University of Huelva (see Tables 1, 2).

**Table 1:** Age of participants.

Age band	18–19	20–21	22–23	24–25	26–30	>30
No. of participants	40	57	20	4	4	3

Six age intervals were established for classifying the participants.

**Table 2:** Gender of participants.

MALE: 37 participants	FEMALE: 91 participants	OTHER: 0 participants

Participants self-identified their gender.

Students’ responses were stored in a database, from which each variable was analyzed in turn. The variables were as follows:

- Age; six intervals were established: 18–19; 20–21; 22–23; 24–25; 26–30; >30. The average age was 23.2.
- General level of English; three bands were established: below B1; B1 (non-certified), B1 (certified). 77.8% of the respondents were in possession of B1 certification.
- Gender; three responses were available: male, female, other.

### Instruments and data collection

#### Semi-structured interview

A semi-structured interview was used, hosted in Google Forms, consisting of a series of quantitative questions drawn from the CAAAL questionnaire (Peña-Acuña and Crismán-Pérez, 2021), plus two qualitative open-ended questions which gave participants the opportunity to amplify aspects of the previous questions relating to memory and self-evaluation.

The CAAAL questionnaire had been validated in a previous study (Peña-Acuña and Crismán-Pérez, 2021; see Appendix). It consisted of 13 items, with the following Likert scale options: *not really, to a limited extent, reasonably well, quite a lot, very much so*. It was designed to measure *attitudes to learning* and *linguistic attitudes to L2*.

The two qualitative open-ended questions that were added to the semi-structured interview were the following:

1. ‘Why do you think that repetition of content in Papua improves memorization of English content?’
2. ‘Why do you think that self-evaluation in various aspects (average score in pronunciation, average number of correct answers, correction of pronunciation, incorrect answers in terms of content, grammar or appropriateness) in each scenario in the Papua app improves intrinsic motivation for learning English?’

### Procedure

The study followed a mixed methodology within the framework of a previous six-week experimental teaching intervention with university students in the second semester. The students were provided with a password which gave them access to 9 scenarios at the basic level of the app. They were given time to explore the scenarios and test out the app at their leisure, so as to get to know it for themselves and assess how useful they might find it. At the end of this period, the participants remotely evaluated different aspects of the Papua app in terms of various characteristics and components of the software. Specifically, they completed a semi-structured interview composed of a quantitative questionnaire with two additional qualitative questions available in Google Forms. This enabled the researchers to collect data and learn the students’ assessment of the experience.

The advantage of this sample was their background in teacher education, meaning they were familiar with pedagogical concepts. The results were contrasted with two basic components of the foreign language learning process: memory (Ellis, 1996) and self-evaluation, which influenced oral production skills (Kissling and O'Donnell, 2015). Nevertheless, these results should be compared with other prospective studies, both of apps as learning software and of other non-digital tools for learning a foreign language.

### Data analysis

The analytical procedure took the following methodology. First, all documentation concerning the app provided by its creators was read by the researchers so as to familiarise themselves with the conceptual origins of the app and the features it offered. The researchers then followed this up with two interviews with the creators of the app. The aim was to find out more about the kind of learning its creators hoped to achieve through the app. The outcome of these interviews was the addition of the two qualitative questions to the quantitative questions in the CAAAL questionnaire in order provide a semi-structured interview in accordance with a mixed methodological approach. These open questions focused on the individual components of Papua in relation to the factors included in the quantitative section.

The same 128 participants then individually completed the two-part questionnaire on Google forms while their experience of using the app was still fresh in their minds.

#### The quantitative analysis

Once the responses of the sample had been collected, the quantitative data were analysed using statistical tests in the SPSS package (version 25).

After the questionnaire had been statistically fit to the sample in question, Cronbach’s alpha was over 0.7, while the KMO index was 5.1, with a significance of 0.001 with respect to these two variables (attitudes to learning and linguistic attitudes to L2). It was administered online *via* Google forms at the end of the six-week intervention period during the academic year 2020–2021.

These variables were contrasted with the responses to the dimensions below, giving particular focus to those concerning memory and self-evaluation. In addition, questions 5, 8, 10, 11, 12 and 13 in the CAAAL questionnaire (Peña-Acuña and Crismán-Pérez, 2021) were considered. The full list of dimensions is as follows:

- Oral competences
- Reading competence
- Linguistic communication (interpretation and communication feedback).
- Cognitive knowledge facilitators (memory)
- Self-evaluation report (response rating for each content unit).

#### The qualitative component

In addition, regarding the qualitative analysis of the collected data, an initial analogical analysis of the components was carried out by the researchers. Finally, when the components for each question were available, the analysis of the qualitative data was carried out using the QDA Miner program, version 2.0.8. This program allows categorization into components, subcomponents and descriptors. The main advantage was that the program allowed the use of qualitative data alongside the quantitative frequency of the subcomponents used by the respondents, enabling a percentage hierarchy of standout terms in the sample to be established.

The advantage of incorporating a qualitative dimension in the study is that it enables the topic to be studied in greater depth, with a wide range of variables, some expected and others emergent. These data are of a different, but complementary, nature. While the quantitative data place the emphasis on the objective description of phenomena external to the individual, the qualitative perspective interprets the subjectivity of the agents involved, along with those phenomena resulting from this interaction (Castro, 1996, 64).

The qualitative study was carried out following the eight “big-tent” criteria of rigor and high quality expressed by Tracy (2021, 178), namely: methodological qualitative research tackling a worthy topic, rich rigor, sincerity, credibility, resonance, significant contribution, ethics and meaningful coherence.

## Results

### Quantitative results

With respect to the quantitative results, the variable *level of English* correlated positively with the variable *oral competence* (r = 0.36; *p* < 0.05; r = 0.05 *p* < 0.01). This result was corroborated by a one-factor Anova for the variable *certification of English* (B1 as minimum level) in relation to oral competence where *F* (3,215) *p* = 0.25. The Anova test also gave a result for the cognitive facilitator, *memory F* (2,937) *p* = 0.36. These data indicate that were H_1_, H_2_ y H_3_ fulfilled.

The variable *age* did not display any significant correlations with the other variables. However, the Anova test presented a significant relation *F* (892) *p* = 0.44.

The variable *gender* (feminine) displayed a significant correlation with the variable *level of linguistic communication* (*r* = 0.94 *p* < 0.05).

### Qualitative results

#### First part. Cognitive factor: Memory

The starting question was: ‘Why do you think that repetition of content in Papua improves memorization of English content?’ The starting subcomponents about which it was intended to deepen before doing the semi-structured interview were the following:

- Subcomponent: emotion favoring memory retention.
- Subcomponent: content repetition favoring memory retention.

The results of the semi-structured interview indicated three aspects worthy of note. The first of these was the perception that the app had a positive effect on users’ memorization of content. The second was that participants underlined the situational approach favored by the app, along with other positive features, while the third was an overall constructive response to the app, with a minority of negative comments.

Returning to the first – the perception of the beneficial effects of repetition on memorization – this was the predominant factor across the user sample (56.7%), providing support for with H_3_. The respondents reported that this memorization was achieved by means of understanding material delivered *via* the oral, visual, written, spoken and aural expressive manifestations (6.8%); paying attention to error correction (1%) in order to improve pronunciation (4.5%); and engaging with the metacognitive resources (0.3%) to check their own learning, in line with H_4_, H_5_ and H_6_.

With regard to the second aspect – the positive features of the app – the elements most frequently identified were the incorporation of gamification and the situational approach (12.3%) by means of varied resources (activities, dialogues, laboratory, translation, etc.), and learning through storytelling (1.5%). Finally, the application received an overall positive rating (4.5%) and was considered to provide motivation for learning (5.8%).

Within this third aspect were to be found critical appraisals, from both negative and constructive perspectives, concerning certain structural aspects of the app of less significance.

#### Second part. Improving intrinsic motivation through self-evaluation

The starting subcomponents about the self-evaluation before passing the qualitative question of the semi-structured interview consisted of this list of items:

- Average score in pronunciation.
- Average number of correct answers.
- Incorrect answers (content errors).
- Incorrect answers (grammatical errors).
- Incorrect answers (socio-culturally inappropriate).

The starting question in the semi-structured interview was: ‘Why do you think that self-evaluation in various aspects (average score in pronunciation, average number of correct answers, correction of pronunciation, incorrect answers in terms of content, grammar or appropriateness) in each scenario in the Papua app improves intrinsic motivation for learning English?’

Three main aspects emerged from the qualitative analysis of the responses to this question. Firstly, respondents noted the positive attributes of the app. Secondly, as a constant in the results of the previous question, they highlighted the positive qualities of the app and other positive aspects. Thirdly, they expressed both constructive and negative critical appraisals.

With respect to the first of these aspects, the predominant attribute identified was motivation (54.4%) followed by learning progress (25.8%). It is important to highlight that respondents perceived these two attributes as connected, sometimes causally, sometimes correlatively. Other attributes which emerged included the ability to personalize the app (9.4%), and the self-evaluation function (2.4%).

The second aspect highlighted positive adjectives applied to the app (6.7%).

The third aspect concerns critical appraisals regarding the self-evaluation function (0.9%), including a negative one (0.3%), although these positions were in the minority.

The third component refers to more critical (0.9%) and even negative (0.3%) positions in the minority regarding the self-assessment element in the application.

## Discussion and conclusion

As will be shown below, both the general and specific aims of this study were fulfilled. Regarding the first research question, concerning the perception of an improvement in learners’ memorization of vocabulary using Papua, the study provided support for the findings of previous studies into MALL methodology (Han, 2015; Han et al., 2017; Wang, 2019). In short, with respect to the first research question, an improvement in vocabulary retention was perceived in the following specific respects:

- The reading activities in the app boost memorization of words.
- Taking a role involving the expression of one’s emotions improves the assimilation of English vocabulary.
- Activities involving repetition of material, or a spiral syllabus, improve memory capacity.

The findings from the study are similar to those of other research in the field. Barrett et al. (2021), investigated the attitudes of 30 university students towards using app called EOPA for oral presentations in English. In this case, 37% reported a positive attitude, while 33% reported having experienced difficulties in using it.

A meta-analysis carried out by Lin and Lin (2019) of 33 quasi-experimental studies published between 2005 and 2018 found an overall positive attitude towards both mobile applications and the use of MMS/SMS for language learning. This latter modality proved to be most effective with regard to retention of words and other lexical units in L2. The meta-analysis also revealed that there remain relevant areas to be researched in the field, including the settings in which the research takes place, the autonomy such applications confer on the user, and the duration of the experimental periods. Rosell-Aguilar (2018) demonstrated that mobile teaching applications improved L2 learning especially with regard to lexis, in terms of the memorization of words.

With regard to the third research question – the issue of whether Papua favors an improvement in oral competences above the written – our findings concurred with those of Shen and Park (2020), who studied the relationship between working memory and English language learning by Chinese students using MALL methodology over the last 20 years. Their findings showed that oral competences improved more than other dimensions involved in second language learning.

Our study of Papua likewise indicated that the app positively impacted oral competences more than it did other competences and dimensions involved in learning English (Yi and Luo, 2012; Yi and Ni, 2015). In this respect, we would maintain that the use of artificial intelligence for voice recognition is a key function of the app, and contributed to the improvement in users’ pronunciation. There is, however, a need to confirm the validity of this finding through future research into the effect of such apps on the attentional component of working memory (see Tables 3, 4).

**Table 3:** Improving memorisation of content.

Components	Subcomponents	Descriptors
Memory retention	Repetition and learning of content 56.7%	Vocabulary, expressions, phrases, grammatical structures, helps internalize, recognize, associate, strengthen neural connections, reinforce visual and auditory memory, in written and spoken form
	Comprehension 6.8%	Content understood in relation to practice
	Pronunciation 4.5%	Improvement in pronunciation, recognising pronunciation
	From errors 1%	Dichotomy correct form/error, learning from errors
	Metacognitive resource 0.3%	Learning checks
Qualities of the app	Situational learning 12.3%	In a natural context, everyday context, learning to communicate in English; conversations set everyday situations; based on communicative situations; through various resources and activities, revises material through a choice of different ways; activities, dialogues, learning laboratory; you learn behavior through sounds, words and pictures
	Motivating 5.8%	Not monotonous, you do not get bored, it grabs your attention, it gets you hooked, easy, nice
	Gamification 5.3%	Game-based, involves games
	Positive qualifiers about the app 4.5%	Fantastic, brilliant, original, different, new, dynamic, fun, entertaining, complete, innovative, useful, interactive, effective
	Use of storytelling 1.5%	Story, narrative, episodic stories, divided into scenes; a real story in which we take the lead role
Critical attitude towards the app	Negative 0.8	It’s badly thought out in this respect, as it makes us look at the same terms several times; so much repetition becomes tiresome
	Constructive 0.5%	I’d add more scenes and more varied activities at each level; I’d use visual images for memorising words; alternate different games at the end of each scene

**Table 4:** Improving intrinsic motivation through the app’s self-evaluation module.

Components	Subcomponents	Descriptors
Positive functions of the app’s self-evaluation module	It motivates the user 54.4%	It allows the user to keep making progress and motivates them, they do not feel the pressure of an exam or a classroom situation; there is intrinsic motivation; it’s motivating; it makes you concentrate at the higher levels; your self-esteem goes up; it gives you recognition; it makes us feel proud of ourselves; it makes you demand a lot of yourself; it makes learning English like a game; it makes you push yourself; you enjoy learning
	It encourages learning progress 25.8%	Students receive different tips for improving; it makes you work harder; it helps to improve your social interaction, pronunciation, writing, reading comprehension, awareness of the other culture, knowledge of the language, the sentence structures help you gain fluency in English; it helps you to overcome embarrassment and difficulties in pronunciation
	Personalization of learning 9.4%	People who are shy or get embarrassed easily might it suits them; you can identify with it; it makes you feel secure; it takes the user’s decisions into account; you can go at your own pace and level; it lets you analyse and assess your strong and weal points; you learn from your errors; it gives you autonomy and self-direction; active agent; it encourages self-reflection and feedback; it makes us focus more on where we need to improve; it lets you learn on your own; having more confidence in the things you do right
	Self-evaluation: outstanding feature 2.4%	You find out where you went wrong; it’s very detailed and precise; it’s even more important in this app; it gives you scores and feedback; the parameters it measures are very interesting; it focuses on what you do right and gives you recognition for this
Qualities of the app	Qualifiers about the app 6.7%	It’s very original; a different learning tool; innovative, interactive; suitable and fun; useful; game-based, visual and efficient; interesting
Critical attitude towards the app	Constructive 0.9%	If it corrected you immediately or explained what the cause of the errors you made was in the test at the end of each stage; it ought to give you the chance to listen to the different opinions first and then give the answers by speaking; it should evaluate you a different way, explaining where and why you went wrong
	Negative 0.3%	The app has positive features, but this does not necessarily mean it boosts motivation; it does not seem to me to be very accurate in its pronunciation scores

With respect to the second dimension considered by this study – the relationship between self-evaluation and users’ intrinsic motivation – the results of the study confirm the second and fourth research question. That is, through the incorporation of SE, the app successfully provided intrinsic motivation, monitoring of progress and personalization of learning. The findings confirmed the relationship between gamification and student motivation (Dehghanzadeh et al., 2021), as analysis of the answers from the semi-structured interview indicated.

These results are consonant with those of a study carried out by Yeh and Chen (2019), which found that the SE component of the language learning app under consideration had most impact on students’ performance and motivation, irrespective of gender. Likewise, a study into the effect of MDA on learners’ spoken accuracy carried out by Rezaee et al. (2019) concluded that MDA positively influences performance. Consequently, our study can be placed alongside those such as Cahyono and Ludwig (2017), and Chin-Hsuan et al. (2021), which provide evidence for the positive benefits of MDA for English language learning.

It can be seen from the literature review that professionals in field have a broadly positive view of SE as an educational and metacognitive tool, with the monitoring of progress, attention to learning strategies, and learner autonomy all features which have been foregrounded. In this respect, García Botero et al. (2021) conclude that learners who receive training and scaffolding for self-regulation within a MALL context benefit more from using the language learning platform. Nevertheless, some possible criticisms can be noted.

In the qualitative phase of the study, the respondents foregrounded intrinsic motivation, thus providing support for the following findings corresponding to the second and fourth research question:

- Self-evaluation in terms of average scores in pronunciation and answers for each scene boosts users’ intrinsic motivation for learning English.
-Self-evaluation in terms of wrong content-based answers in each scene boosts users’ intrinsic motivation for learning English.
-Self-evaluation in terms of wrong socio-cultural answers in each scene boosts users’ intrinsic motivation for learning English.

Further, it is worth noting that monitoring progress and personalization were also found to be significant factors, albeit to a lesser degree. Although monitoring progress has become an area of continued educational interest, as evidenced by the increase in the number of patents filed for related technology, there has also been some skepticism towards SE on the basis of mismatches between the descriptors and students’ own perceptions (García Sanz, 2020). Similarly, a review of the literature shows that personalized learning *via* technology is highly regarded among educationalists, and is one of the most studied resources for language learning (Zhou et al., 2016), prompting a comparable deluge of patents (Chao et al., 2009). The overwhelming interest in the potential of these tools is what led us to research their effectiveness in the L2 learning process, with the aim of identifying positive common results, and complementarily, areas and aspects for improvement.

### Future research

As noted above, the assessment of the Papua app carried out by this has been largely positive. Nevertheless, some drawbacks were noted in the course of the research, as well as among the findings, and these will be considered below.

First of all, as García Botero et al. (2019) notes, despite the self-directed learning capabilities of this kind of tool, the provision for training and scaffolding is nevertheless to be highly recommended. This would take the form of some kind of tutorial or partially in-person course to ensure that users get maximum benefit from its use. Otherwise, there is a high risk of learners becoming demotivated.

In addition, it would be interesting to complement the research presented in this paper with a study into the use of the app as a long-term methodological component in a language learning course, as opposed to the short-term (6-week) period which formed the basis of this study. As Shantanu Tilak et al. (2021) point out the majority of studies tend to focus on the design and evaluation of the tool under analysis itself, with far less attention paid to assessing the sustained effectiveness of such digital apps. The findings of this study should thus be treated with caution, until such a time as a longitudinal study can be carried out to explore the possibilities of this tool in particular, and the use of MALL methodology in general, as part of the everyday language learning resources.

In like manner, the current study focused on vocabulary retention by virtue of its link to memory. Nevertheless, given that the tool allows the enactment effect to be incorporated, we believe it would be useful to undertake complementary studies to explore the improvement of the user’s pronunciation on the phonemic plane, as well as their grammatical knowledge. Such studies would enable us to assess the effectiveness of the tool over a range of language skills, and would allow the traditional cognitive monitoring processes applied in this tool to be compared with other tools and methodologies for language learning. In this respect, the work of Verhagen et al. (2015) and Serafini and Sanz (2015) is worthy of note, as both studies found a correlation between phonological and grammatical memory. This kind of directed research, exploring the connections between different modules involved in the language learning process, would help Papua to be developed so as to take account of the different responses of the sample according to their intrinsic characteristics in terms of age, level of English, and gender. Likewise, another function that we have considered, taking into account the significant relationship between working memory and the memory for the oral competences and vocabulary retention, is the addition of an instrument for measuring working memory, along with an alternative methodology for increasing this kind of memory.

It is also worth noting that, as Ni (2018) and Shen and Park (2020) maintain, working memory and vocabulary storage in learning English as foreign language are correlated, something which is equally true at the initial stages of learning English as a foreign language (Li, 2004; Jiang et al., 2015). This leads us to the conclusion that it is important to consider memory as a major dimension within the field of language learning, and more specifically, the development of working memory for its interrelation with language learning and in itself.

As noted above, the study described here threw up an emergent variable in the shape of storytelling. This was identified as aiding both memory and motivation while learning English in a MALL context. This variable could fruitfully be the subject of a research question in future studies exploring its influence on the effectiveness of this kind of digital tool.

Finally, we also need to be aware that our study focused on a sample of students whose first language was Spanish. If we are to develop a broader and deeper theory of the advantages of using digital tools such as the Papua app, it will be necessary to take account of a wider range of language backgrounds. In similar vein, it would be interesting to broaden the range of languages available to be learnt on the app so as to note whether the findings regarding working memory and SE were consistent with those presented here. This would perhaps allow us to make more general statements about cognitive aspects such as the connections between memory and language learning, and at the same time establish templates for this kind of learning consistent with methodologies like SE.

We should also be aware of the absence of any measurement of affect in the study. As Arnold (2000) points out, affect is one of the key factors influencing second language learning due to its role in determining the learner’s attitude towards the language and towards their perception of success, beyond any other factors involved in the process. This raises a new issue for MALL methodology, namely, how to make up for the reduced levels of affect resulting from learners working in isolation. As we suggested above, one means might be to complement this kind of learning with some degree of in-person input or tutoring, but it remains, we think, an interesting question to be tackled in future studies.

At this point, in prospective studies we also intend to examine the relationship between the self-regulation of learning/acquisition of a foreign language/L2 from a digital tool such as Papua and a possible correlation with the motivation variable, which is linked to the plane affective behavior of students’ attitudes (Arnold, 2000).

For this we must start again from the traditional EFL learning/acquisition strategies (Oxford, 1990). However, it is necessary to review these strategies according to a digital learning framework, due to the latest innovations in software for the learning/acquisition of foreign languages/L2 and the new demands and needs of students, especially noticeable since COVID-19. and the EFL virtual learning/acquisition methodology.

## Data availability statement

The original contributions presented in the study are included in the article/supplementary material, further inquiries can be directed to the corresponding author.

## Ethics statement

Ethical review and approval was not required for the study on human participants in accordance with the local legislation and institutional requirements. The patients/participants provided their written informed consent to participate in this study.

## Author contributions

Conceptualization and investigation, BPA and RCP; methodology and formal analysis, BPA and RCP; Writing – Original Draft, BPA and RCP; Writing – Review & Editing, BPA and RCP. All authors read and approved the final manuscript.

## Funding

This study received funding from Aprendizaje inmersivo S.L. (2021). The funder was not involved in the study design, collection, analysis, interpretation of data, the writing of this article or the decision to submit it for publication. All authors declare no other competing interests.

## Conflict of interest

This study received funding from Aprendizaje inmersivo S.L. The funder had the following involvement with the study: funding to promote scientific research. All authors declare no other competing interests.

## Publisher’s note

All claims expressed in this article are solely those of the authors and do not necessarily represent those of their affiliated organizations, or those of the publisher, the editors and the reviewers. Any product that may be evaluated in this article, or claim that may be made by its manufacturer, is not guaranteed or endorsed by the publisher.

## Appendix

Quantitative questionnaire on language attitudes and learning attitudes towards the Papua tool.

1. Do you think that the activities included in the app boost fluency in informal communication as a result of encouraging interaction in real, contextualized social situations?

Not really To a limited extent Reasonably well Quite a lot Very much so.

2. Do you think that the activities included in the app boost oral comprehension in informal communication as a result of encouraging interaction in real, contextualized social situations?

Not really To a limited extent Reasonably well Quite a lot Very much so.

3. Do you think that the activities included in the app boost self-confidence in speaking English in informal communication as a result of encouraging interaction in real, contextualized social situations?

Not really To a limited extent Reasonably well Quite a lot Very much so.

4. Do you think that the activities included in the app improve reading comprehension in foreign language?

Not really To a limited extent Reasonably well Quite a lot Very much so.

5. Do you think that the reading activities included in the app improve the memorization of words?

Not really To a limited extent Reasonably well Quite a lot Very much so.

6. Do you think that the activities included in the app improve users’ morpho-syntactic knowledge of foreign language?

Not really To a limited extent Reasonably well Quite a lot Very much so.

7. Do you think that the actors’ emotional performances in the real-life scenes included in the storyline are influential in improving users’ foreign language vocabulary?

Not really To a limited extent Reasonably well Quite a lot Very much so.

8. Do you think that taking a role which involves expressing your emotions helps you to memorize foreign language?

Not really To a limited extent Reasonably well Quite a lot Very much so.

9. Do you think that taking a role which involves expressing your emotions improves your assimilation of foreign language vocabulary?

Not really To a limited extent Reasonably well Quite a lot Very much so.

10. Do you think that activities involving repetition of material or a spiral syllabus improve memory capacity?

Not really To a limited extent Reasonably well Quite a lot Very much so.

11. Do you think that self-evaluation in terms of average scores in pronunciation and answers for each scene boosts users’ intrinsic motivation for learning foreign language?

Not really To a limited extent Reasonably well Quite a lot Very much so.

12. Do you think that self-evaluation in terms of wrong content-based answers in each scene boosts users’ intrinsic motivation for learning foreign language?

Not really To a limited extent Reasonably well Quite a lot Very much so.

13. Do you think that self-evaluation in terms of wrong socio-cultural answers in each scene boosts users’ intrinsic motivation for learning foreign language?

Not really To a limited extent Reasonably well Quite a lot Very much so.
